# Physiotherapy case reports on three people with progressive supranuclear palsy

**DOI:** 10.3389/fnagi.2023.1294293

**Published:** 2023-12-08

**Authors:** Mariana Mateus, Alexandre Castro Caldas

**Affiliations:** ^1^NeuroSer, Lisbon, Portugal; ^2^Centro de Investigação Interdisciplinar em Saúde—Universidade Católica Portuguesa, Lisbon, Portugal

**Keywords:** progressive supranuclear palsy, physiotherapy, falls, functionality, quality of life

## Abstract

**Introduction:**

Progressive supranuclear palsy (PSP) is a neurodegenerative brain disease that affects patient’s functionality and quality of life. Physiotherapy should be recommended at the time of diagnosis to slow the progression of disability and enhance the quality of life of these patients.

**Clinical presentation:**

Here, we describe three cases of patients with PSP, outlining their motor and non-motor symptoms and examining their clinical progression with physiotherapy intervention. During the initial intervention years, a reduction in the number of falls was achieved, along with improvements in gait and balance.

**Conclusion:**

Exercise and physiotherapy appear to be beneficial for patients with PSP by enhancing their functionality and quality of life. Controlling or reducing the number of falls should be the primary goal of any intervention for patients with PSP.

## Introduction

In recent years, there has been a significant increase in research examining exercise interventions and their benefits for neurological diseases (Radder et al., 2020; Bliss et al., 2021; Ellis et al., 2021; Guadarrama-Molina et al., 2021; Johansson et al., 2022; Okemuo et al., 2023). Neurological diseases involve various motor changes, such as balance issues, increased risk of falls, muscle weakness, movement impairments, coordination changes, gait changes, and consequently reduced functional independence and lower quality of life (Okemuo et al., 2023).

Physiotherapy, a non-pharmacological intervention involving specific exercises, improves the motor function and quality of life of these patients (Radder et al., 2020; Costa et al., 2023; Tobar et al., 2023). Evidence indicates positive effects of exercise on brain health (Cardoso et al., 2022) maintaining optimal cerebrovascular function, and potentially preventing cognitive impairment (Bliss et al., 2021).

In the case of Parkinson’s disease (PD) and Progressive supranuclear palsy (PSP), physiotherapy should be recommended at the time of diagnosis to slow the progression of disability and enhance the long-term quality of life of patients with these diseases (Slade et al., 2019, 2020; Ellis et al., 2021; Costa et al., 2023). Exercise alleviates both motor and non-motor symptoms (Johansson et al., 2022; Costa et al., 2023).

Progressive supranuclear palsy is a progressive neurodegenerative brain disorder, the most frequent form of atypical Parkinsonism, characterized by nerve cell degeneration mainly in the brainstem (Höglinger et al., 2017). The diagnosis of PSP is definitively determined through neuropathological examination, revealing the accumulation of intracerebral aggregates of the microtubule-associated protein tau (4R-tau) in neurofibrillary tangles, oligodendrocyte coils, and astrocytic tufts (Barsottini et al., 2010; Höglinger et al., 2017). This results in neurodegeneration and gliosis in the basal ganglia, brainstem, prefrontal cortex, and cerebellum (Clerici et al., 2017). PSP has traditionally been considered a sporadic disease, consistent with genetic associations with markers on chromosome 17q21 and reports of familial PSP cases suggest family aggregation (Barsottini et al., 2010).

The incidence of PSP begins at the age of 40 years or later with an estimated prevalence of 6–10% (Barsottini et al., 2010), an annual prevalence of 5–7 cases per 100,000 persons (Shoeibi et al., 2019; Barer et al., 2022; Swallow et al., 2022), and an annual incidence density rate between 0.16 and 2.6 per 100,000 persons (Stang et al., 2020; Viscidi et al., 2021; Lyons et al., 2023), both increasing with age (Houghton and Litvan, 2007). The average life expectancy after diagnosis is 5–8 years (Steffen et al., 2015; Barer et al., 2022).

The clinic-pathological variants of PSP present with different severities, regions of pathological involvement, and clinical features (Barsottini et al., 2010; Höglinger et al., 2017). Diagnosis is often challenging, and the International Parkinson and Movement Disorder Society (MDS) clinical criteria provide expert guidance (Höglinger et al., 2017). Patients exhibit various symptoms, including vertical supranuclear gaze palsy, postural instability, history of falls (often backward), bradykinesia, rigidity, gait disorders, freezing, speech problems, swallowing difficulties, and cognitive dysfunction (Barsottini et al., 2010; Clerici et al., 2017; Höglinger et al., 2017; Croarkin et al., 2023). Falls are a major concern, leading to reduced independence, morbidity, and mortality (Morris et al., 2015; Clerici et al., 2017). Consequently, patients may have difficulty seeing obstacles and curbs while walking and going up or down stairs, and judging distances while scanning the environment (Steffen et al., 2015).

Non-motor symptoms, such as fatigue, apathy, impulsivity, anxiety, and depression, are common in PSP patients (Steffen et al., 2015; Morris et al., 2021). While there is no cure for PSP, pharmacological therapy is the first approach, and a multidisciplinary team and palliative methods are often required in the later stages (Barsottini et al., 2010; Morris et al., 2021). Rehabilitation, including physiotherapy, is crucial for improving gait and preventing falls, hallmarks of the disease (Slade et al., 2019, 2020; Wittwer et al., 2019; Morris et al., 2021).

However, evidence of the long-term benefits of physiotherapy, especially for advanced PSP, and the most effective forms of exercise, structured activities, and rehabilitation treatments remain insufficient (Clerici et al., 2017; Slade et al., 2020). This case series aimed to contribute to the understanding of PSP by describing three cases and their clinical evolution following physiotherapy.

## Case reports

### Case 1

In 2016, a 77-year-old man received a diagnosis of PSP, although initial symptoms began in 2008. He had no family history of the disease or significant co-morbidities. Non-motor symptoms included anxiety, which was primarily related to changes in daily routines and the onset of freezing episodes. Cognitive changes were absent during the initial years but were identified after the COVID-19 isolation period.

In his daily life, he consistently walked with the support of another person due to the increased risk of falls. Physiotherapy commenced in April 2016 and involved two sessions per week, each lasting 1 h, for 3 years. During the initial assessment, the patient stated his main problems as “starting to walk, moving in the morning and falling many times during the day.” Conversely, his daughter noted increased falls, decreased balance, and reduced strength as the main concerns.

The patient was highly motivated, attended sessions regularly, and had robust family support, all of which positively contributed to the intervention. The initial examination revealed global bradykinesia, muscular stiffness, postural instability (with a history of falls eight times per week), gait disorder (decrease in cadence, step length, stride length, velocity, and step width) in the presence of freezing episodes, balance impairment, weakness, and postural changes, moderately affecting independence, daily life activities, and quality of life.

Physiotherapy was adapted over time to suit the patient’s evolving needs and limitations, encompassing both the type and intervention exercises. The sessions included various exercises, such as dual-task gait training, music-cued movement, balance exercises, resistance strength training, treadmill training, aerobic exercises, fall education, functional mobility training (sit-to-stand, mobility in bed, going up, and down stairs), boxing, and dancing. Family involvement was significant, with sessions aimed at teaching strategies to safely manage freezing episodes, providing safe walking support, and sharing fall prevention strategies.

During the first two 2 years of physiotherapy (Table 1), notable improvements were observed in balance [Berg Balance Scale (BBS): 23 → 39/56; Push and Release Test: 4 → 3], gait [Timed Up and Go Test (TUGT): 11.33 s → 8.87 s; Dynamic Gait Index (DGI): 9 → 16], and strength [Five Times Sit to Stand (FTFTS): 13.16 s → 12.94 s]. Falls were significantly reduced to one per week, primarily due to freezing episodes [Freezing of Gait Questionnaire (FOGQ): 13 → 10]. The patient regained the ability to walk independently and perform daily activities, which contributed to improved socialization and quality of life.

**Table 1:** Scores of scales over time during the intervention.

	First assessment			First revaluation (3 months after first)			Second revaluation (1 year after first)			Third revaluation (2 years after first)			Fourth revaluation (3 years after first)			Fifth revaluation (4 years after first)
Scales	C1	C2	C3	C1	C2	C3	C1	C2	C3	C1	C2	C3	C1	C2	C3	C3
BBS	23/56	24/56	43/56	38/56	35/56	56/56	39/56	38/56	54/56	31/56	35/56	43/56	27/56	18/56	34/56	40/56
Push and Release Test	4	4	1	2	3	2	3	3	3	4	3	4	4	4	4	3
TUGT (seconds)	11.33	14.52	7.44	8.86	12.88	7.16	8.87	20.52	6.81	8.67	23.59	8.34	11.36	24.95	8.39	8.24
FOGQ	13	-	0	9	-	12	10	-	18	16	-	20	16	-	23	16
History of falls (Per week)	2–3	2–3	No history of falls.	1	1	3	1	2	5	2	1	6–8	3	0	6–8	6–8
UPDRS-III	46	32	25	38	39	12	58	61	30	67	59	49	82	68	71	71

C1, Case 1; C2, Case 2; C3, Case 3; BBS, Berg balance scale; TUGT, Timed up and go test; DGI, Dynamic gait index; FTFTS, Five times sit to stand; FOGQ, Freezing of gait questionnaire; and UPDRS-III, Unified Parkinson’s Disease Rating Scale (Part III).

The patient experienced an anticipated decline in motor function during the final year of physiotherapy, as evidenced by an increase in Unified Parkinson’s Disease Rating Scale—Part III (UPDRS III): 46 → 82. Despite disease progression, the patient maintained an independent gait with supervision and support for daily activities. Speech and swallowing difficulties emerged, leading to additional home-based speech therapy.

Physiotherapy was interrupted in March 2020 owing to the COVID-19 isolation period, resulting in a significant exacerbation of sedentary behavior. Post this period, the patient continued physiotherapy at home but unfortunately succumbed to a respiratory infection caused by prolonged hospitalization in 2021.

### Case 2

In 2016, a 72-year-old woman received a PSP diagnosis, with the initial symptoms emerging in 2013 following the tragic death of her grandson. She had no family history of the disease but had controlled hypertension, anxiety, and depression as co-morbidities.

In terms of non-motor symptoms, she presented with anxiety, apathy, and fatigue but no cognitive changes. In her daily life, she consistently walked with the support of her husband, and at home, she used a walker.

Physiotherapy commenced in September 2016, involving two sessions per week, each lasting 1 h, for 3 years. During the initial assessment, she expressed her main concern as “balance, walking, fear of falling, and fatigue.” Conversely, her husband who served as a caregiver, identified sitting/standing and balance as her main problems.

The patient demonstrated motivation during the physiotherapy sessions but exhibited negativity regarding her clinical condition, leading to increased sedentary and apathetic behavior. Conversely, her positive and humorous husband contributed to her motivation, facilitating her continued movement.

During the initial examination, the patient displayed widespread bradykinesia, muscular stiffness, and gait disorders characterized by reduced cadence, step length, stride length, velocity, and step width. She also demonstrated balance impairment, overall weakness, and postural changes. A sedentary lifestyles and decreased tolerance for exertion were identified as significant challenges. The physiotherapy intervention was tailored to her fatigue levels and included various exercises, such as gait training with a dual-task, music-cued movement, balance training, resistance strength training, treadmill training, aerobic exercises, fall prevention education, functional mobility training (sit-to-stand, mobility in bed, up, and down stairs), boxing, and dancing. Several family sessions were conducted to encourage home exercises, provide for managing freezing episodes, along with safe walking practices.

Similar to the first patient, during the first 2 years of physiotherapy (Table 1), she demonstrated significant improvement balance (BBS: 24 → 38/56; Push and Release Test: 4 → 3). For safety reasons, she could walk only with supervision, and some activities of daily living still required assistance. There was a notable enhancement in her mood and engagement in day-to-day life, as she felt more secure in performing tasks.

During the final year of physiotherapy, the anticipated deterioration of her motor condition (UPDRS III: 32 → 68) led to an increased sedentary lifestyle and a greater degree of dependence. These factors have prompted the transition to home-based physiotherapy. Additionally, the worsening dysphagia condition led to the placement of a percutaneous endoscopic gastrostomy (PEG).

Unfortunately, she passed away in March 2021 after developing aspiration pneumonia.

### Case 3

In 2016, a 68-year-old woman was diagnosed with PSP. Her initial symptoms, primarily manifested as reduced gait speed, began in 2014. She had no family history of the disease but had anxiety as a comorbidity. Residing with her 101-year-old mother, she received full-time support from a caregiver.

Physiotherapy commenced in June 2017, with two sessions per week, each lasting 1 h. During the initial assessment, she identified her main problems as “balance, walking slowly, and fatigue.” The patient was highly motivated and committed to the sessions, but was also impulsive and anxious, negatively impacting her motor condition and daily life activities and exposing herself to risky situations.

During the initial examination, the patient exhibited widespread bradykinesia, muscular stiffness, and gait disorder characterized by decreased cadence, step length, stride length, velocity, and step width, along with freezing episodes. Balance impairment, overall weakness, and postural changes were also observed. The frequency of physiotherapy sessions varied over the years: two sessions per week in the first year, three in the second, five in the third year, and currently four. These sessions are supplemented by weekly cognitive stimulation and occupational therapy sessions. Cognitive stimulation aims to enhance overall cognitive function, whereas occupational therapy promotes autonomy and independence in activities of daily living.

Multidisciplinary intervention has proven to be fundamental in enhancing at patient’s overall function, with objectives defined collaboratively by the three areas involved. Physiotherapy interventions include various exercises such as gait training with dual-task, music-cued movement, balance exercise, resistance strength training, treadmill training, aerobic exercises, fall prevention education, functional mobility training (sit-to-stand, mobility in bed, up, and down stairs), boxing, and dancing. Family and caregiver sessions were conducted to teach strategies for dealing with freezing episodes and ensure safe walking practices.

Consistent with the progress observed in the first two patients, during the initial 2 years of physiotherapy, this patient showed improvement in balance (BBS: 43 → 54/56) and in strength, with enhancements in walking parameters such as velocity, cadence, and safety (Table 1). These improvements enabled independent walking sessions and enhanced the safety of daily life activities.

The 3-month interruption of physiotherapy sessions due to COVID-19 resulted in a significant increase in sedentary lifestyle and physical inactivity. Video calls were employed during this period to guide patients in safe home exercises. Upon returning to the clinical sessions, there was a worsening in the duration and frequency of freezing episodes, with the emergence of festination, impacting speech and writing. The patient required continuous assistance for safe walking.

Currently, the primary concern is the frequency of freezing episodes, particularly when changing direction, which poses a safety hazard and increase the risk of falls. The patient’s impulsivity and reluctance to fully accept her condition put herself at risk. Despite this, the patient has retained the remaining motor capabilities. Although her posture deteriorated, she is still able to walk without the need for supervision.

The families of patients in cases 1 and 2 authorized the use of their family members’ data in this study, and patient 3 provided a written informed consent to participate.

## Discussion

Physiotherapy interventions for PSP face challenges owing to the absence of clinical guidelines on exercise for this disease. Previous studies have emphasized the importance of early exercise for enhancing to strength and overall motor function, particularly before cognitive impairment occurs (Wittwer et al., 2019; Morris et al., 2021; Dale et al., 2023). Case 3 highlights the benefits of initiating physiotherapy during the early stages of the disease.

Sustained and intense exercise positively affects brain function and structure (Frazzitta et al., 2014; Liu et al., 2019; Johansson et al., 2020). Case 3’s 6-year intensive intervention may provide insights unto the benefits of such exercises on disease progression. Multimodal training is necessary due to the diverse motor functions affected by PSP, which aligns with the importance of walking programs and balance training (Clerici et al., 2017; Wittwer et al., 2019; Slade et al., 2020).

Adapting interventions to the individual needs, preferences, motivations, and involvement of caregivers is crucial for continued intervention at home and, promoting greater knowledge about the disease and coping mechanisms (Slade et al., 2019, 2020). Communication difficulties and cognitive impairment can limit social participation (Slade et al., 2020; Morris et al., 2021), as observed during the COVID-19 interruption.

A history of falls poses a considerable challenge in working with individuals with PSP (Canning et al., 2015), as observed in the described cases. Fall prevention exercises, corrective postural reaction training, and eye movement/visual awareness training have demonstrated effectiveness in reducing falls and enhancing overall mobility (Steffen et al., 2015; Dale et al., 2023; Intiso et al., 2023). The three reported cases showed a decrease in falls through the intervention, promoting walking safety and improving the ability to manage freezing episodes. However, impulsivity remains a negative factor influencing the frequency and severity of freezing episodes.

The primary goal of interventions for individuals with PSP should be to minimize or eliminate falls, as evidenced by the positive outcomes observed in the described cases. A multidisciplinary approach and effective communication with neurologists are essential for comprehensive patient monitoring (Clerici et al., 2017).

The strength of this study lies in its detailed description of disease progression through physiotherapy intervention, coupled with objective data on various motor functions over the course of the intervention. Exercise and physiotherapy have clear benefits for individuals with PSP as they, enhance their health and functionality. However, additional longitudinal randomized controlled studies are necessary to gain a comprehensive understanding of their impact on adaptive plasticity and, disease progression, and to determine the most suitable types of exercise for this population.

## Conclusion

Early multimodal physiotherapy can enhance balance, gait, strength, and functionality while reducing the frequency of falls in patients with PSP. Personalizing interventions to meet specific needs and involving families and caregivers are crucial. Therefore, a multidisciplinary approach is essential for managing disease progression. Longitudinal randomized controlled studies are necessary to fully understand the effects of physiotherapy and exercise on disease progression.

## Data availability statement

The original contributions presented in the study are included in the article/supplementary material, further inquiries can be directed to the corresponding author.

## Ethics statement

Ethical approval was not required for the studies involving humans because case repots based on data from the archive. The studies were conducted in accordance with the local legislation and institutional requirements. The participants provided their written informed consent to participate in this study. Written informed consent was not obtained from the individual(s) for the publication of any potentially identifiable images or data included in this article because there are no images in the study.

## Author contributions

MM: Writing – original draft, Writing – review & editing. AC: Writing – original draft, Writing – review & editing.
